# Basal‐like breast cancer engages tumor‐supportive macrophages via secreted factors induced by extracellular S100A4

**DOI:** 10.1002/1878-0261.12319

**Published:** 2018-08-09

**Authors:** Lina Prasmickaite, Ellen M. Tenstad, Solveig Pettersen, Shakila Jabeen, Eivind V. Egeland, Silje Nord, Abhilash Pandya, Mads H. Haugen, Vessela N. Kristensen, Anne‐Lise Børresen‐Dale, Oslo Breast Cancer Research Consortium (OSBREAC), Olav Engebråten, Gunhild M. Mælandsmo

**Affiliations:** ^1^ Department of Tumor Biology Institute of Cancer Research Oslo University Hospital Norway; ^2^ Department of Clinical Molecular Biology (EpiGen) Akershus University Hospital Oslo Norway; ^3^ Institute of Clinical Medicine Faculty of Medicine University of Oslo Norway; ^4^ Department of Cancer Genetics Institute of Cancer Research Oslo University Hospital Norway; ^5^ Department of Oncology Oslo University Hospital Norway; ^6^ Department of Medical Biology Faculty of Health Sciences UiT/The Arctic University of Norway Tromsø Norway

**Keywords:** breast cancer, cytokines, S100A4, tumor microenvironment, tumor‐associated macrophages, tumor–stroma interactions

## Abstract

The tumor microenvironment (TME) may influence both cancer progression and therapeutic response. In breast cancer, particularly in the aggressive triple‐negative/basal‐like subgroup, patient outcome is strongly associated with the tumor's inflammatory profile. Tumor‐associated macrophages (TAMs) are among the most abundant immune cells in the TME, shown to be linked to poor prognosis and therapeutic resistance. In this study, we investigated the effect of the metastasis‐ and inflammation‐associated microenvironmental factor S100A4 on breast cancer cells (BCCs) of different subtypes and explored their further interactions with myeloid cells. We demonstrated that extracellular S100A4 activates BCCs, particularly the basal‐like subtype, to elevate secretion of pro‐inflammatory cytokines. The secreted factors promoted conversion of monocytes to TAM‐like cells that exhibited protumorigenic activities: stimulated epithelial–mesenchymal transition, proliferation, chemoresistance, and motility in cancer cells. In conclusion, we have shown that extracellular S100A4 instigates a tumor‐supportive microenvironment, involving a network of cytokines and TAM‐like cells, which was particularly characteristic for basal‐like BCCs and potentiated their aggressive properties. The S100A4–BCC–TAM interaction cascade could be an important contributor to the aggressive behavior of this subtype and should be further explored for therapeutic targeting.

**Abbreviations**
BC: breast cancer
BCC: breast cancer cell
CM: conditioned medium
EMT: epithelial–mesenchymal transition
EpCAM: epithelial cell adhesion molecule
GM‐CSF: granulocyte–macrophage colony‐stimulating factor
HER2: human epidermal growth factor receptor 2
IF: immunofluorescence
IHC: immunohistochemistry
Luc: luciferase
M‐CSF: macrophage colony‐stimulating factor
PBMC: peripheral blood mononuclear cells
TAM: tumor‐associated macrophage
TCGA: The Cancer Genome Atlas
TME: tumor microenvironment
TNBC: triple‐negative breast cancer

## 1 Introduction

Breast cancer (BC) is the most frequent cancer in women. The prognosis and choice of treatment is heavily dependent on the subtype of BC. The majority of the tumors express hormone (estrogen and progesterone) receptors and/or human epidermal growth factor receptor 2 (HER2), allowing targeted treatment using antihormone or anti‐HER2 therapies. For tumors lacking these receptors, *that is*, triple‐negative breast cancer (TNBC), chemotherapy is the only treatment option, although resistance usually develops. Based on gene expression profiling, most TNBC tumors are defined as basal‐like, having high risk of recurrence, metastases, and poor survival (Sorlie *et al*., 2001), which classifies this subtype as aggressive BC.

During the last years, the biological and therapeutic perspective on cancer has evolved from focusing on tumor cells only, to encompass the profound impact of the tumor microenvironment (TME). TME can influence both cancer progression and therapeutic responses (McMillin *et al*., 2013; Quail and Joyce, 2013), and tumor‐related inflammation is now recognized as a hallmark of cancer (Hanahan and Weinberg, 2011). The tumor's immunological portrait – the presence of tumor‐infiltrating leukocytes, their nature, and the profile of regulatory cytokines – is determinative for BC outcome and therapeutic response (Fridman *et al*., 2012; Loi *et al*., 2013). Good outcome/response is associated with a high fraction of cytotoxic T cells and assisting T‐helper 1 cells, whereas poor outcome/resistance is linked to the dominance of T‐helper 2 cells and tumor‐associated macrophages (TAMs) (Savas *et al*., 2016). TAMs are among the most abundant immune cells in the TME, known to be linked to poor prognosis, especially in basal‐like/TNBC (Leek *et al*., 1996; Zhang *et al*., 2013; Zhao *et al*., 2017). In general, macrophages are main cells in the inflammatory response, where they exhibit high plasticity, being able to polarize into distinct phenotypes in response to external signals. The cytokine profile in the microenvironment is essential for macrophage polarization and, consequently, activity (Pollard, 2004). Two extreme variants of macrophage polarization are known as the classically activated (M1) and the alternatively activated (M2) phenotypes. Rather than acting tumoricidal, like classically activated M1 macrophages, TAMs resemble the M2 phenotype and are protumorigenic, that is, can suppress antitumor immunity, promote angiogenesis, stimulate invasion, and facilitate resistance to therapy (Condeelis and Pollard, 2006; Ruffell and Coussens, 2015). Therefore, depletion or reprogramming of TAMs is considered to be an attractive therapeutic option that already approaches the clinic (Ruffell and Coussens, 2015). Further development of TAM‐directed therapies would benefit from better understanding of the complex mechanisms regulating their fate and functions.

Recently, we have demonstrated that macrophage phenotype and functions can be modulated by factors secreted by aggressive melanoma cells that are stimulated with extracellular S100A4 (Bettum *et al*., 2014). S100A4 is a small, Ca^2+^‐binding protein strongly associated with metastasis and poor prognosis in various cancers (Boye and Maelandsmo, 2009; Mishra *et al*., 2011; Rudland *et al*., 2000) and also involved in inflammatory disorders (Cerezo *et al*., 2011; Grigorian *et al*., 2008). S100A4 is expressed not only in tumor cells, but also in various stromal cells, and secreted into the extracellular space (Bettum *et al*., 2014; Cabezon *et al*., 2007). Both endogenous and extracellular S100A4 have been linked to metastasis. Endogenous S100A4 is known to enhanced cell motility and invasion (Grundker *et al*., 2016), while extracellular, stromal S100A4 has been shown to instigate inflammatory events promoting metastasis (Hansen *et al*., 2014).

In this study, we explored the effect of extracellular S100A4 on breast cancer cells (BCCs) of different subtypes and investigated their further interactions with myeloid cells. We showed that S100A4‐activated BCCs elevated secretion of pro‐inflammatory cytokines. The secreted factors facilitated formation of TAM‐like cells with protumorigenic functions. These results suggest that S100A4 instigates a tumor‐supportive microenvironment, involving a range of cytokines and TAM‐like cells, which is characteristic for the aggressive basal‐like BC.

## 2 Materials and methods

### 2.1 Cell lines

Human BCC lines MDA MB 231 (further referred to as MDA231), MDA MB 468 (further referred to as MDA468), SKBR3, and MCF7 were obtained from American Type Culture Collection, while MA11 was established at the Norwegian Radium Hospital (Norway) (Rye *et al*., 1996). The cells were cultured in RPMI‐1640 medium (MDA468 cultured in DMEM) (Sigma, St. Louis, MO, USA) supplemented with 10% FBS (PAA, Pasching, Austria) and 2 mm GlutaMAX (Gibco, Paisley, UK). The immortalized breast epithelial cell line HMLE was a kind gift from R.A. Weinberg (Whitehead Institute, Cambridge, MA) and cultured as recommended. The human monocyte cell line THP1 was kindly provided by R. Solberg (University of Oslo, Norway) and cultivated in RPMI‐1640 supplemented as above with additional 0.05 mm 2‐mercaptoethanol (Sigma). All cell cultures were maintained at 37 °C in a humidified atmosphere containing 5% CO_2_ and were routinely tested for mycoplasma. GFP‐luciferase (Luc)‐labeled MDA468 cells were generated by transducing the cells with lentivirus carrying a GFP‐Luc construct described previously (Day *et al*., 2009) (kindly provided by Glenn Merlino, NIH, MD**)**.

### 2.2 Primary cultures from BC patient biopsies

Surgical specimens of BC tumors were obtained from patients enrolled in the ‘OSL2’ study, conducted at several hospitals in the Oslo region. Informed and written consent was obtained from all patients, and the project was approved by the South East Regional Committee for Medical and Health Research Ethics (Ref. no. 2007/1125, 2016/433). Fresh tumor tissue was minced into ~ 1‐mm pieces that were put in H14 medium [DMEM/F12 (Gibco), 2 mm GlutaMAX (Gibco), 20 mm HEPES, 100 U·mL^−1^ penicillin and 100 μg·mL^−1^ streptomycin, 250 ng·mL^−1^ insulin, 10 μg·mL^−1^ transferrin, 0.1 nm estradiol, 0.5 μg·mL^−1^ hydrocortisone, 0.15 IU prolactin (all Sigma), 2.6 ng·mL^−1^ sodium selenite (BD Biosciences, San Jose, CA, USA), and 10 ng·mL^−1^ EGF (Peprotech, Rocky Hill, NJ, USA)] supplemented with 2% FBS. Cultures were fed one to three times per week by replacing half of the medium with fresh medium. Cells propagating outward from adherent explants were harvested by trypsination and used for further experiments.

### 2.3 *Ex vivo* cultures from patient‐derived xenografts (PDXs)

One luminal (MAS98.06) and one basal‐like (MAS98.12) PDX were established in‐house and described previously (Bergamaschi *et al*., 2009). All animal experiments were performed according to protocols approved by the National Animal Research Authority and conducted according to regulations of the Federation of European Laboratory Animal Science Association (FELASA). For passaging PDXs, mice were anesthetized with sevoflurane (Baxter, Deerfield, IL, USA) and incision was made above sternum to access mammary fat pad, where a small piece of tumor tissue was placed. To establish *ex vivo* cultures, freshly excised PDXs were rinsed in PBS, necrotic tissue/fibrous layer was removed, and the remaining tissue was minced, transferred to a Falcon^®^ 70‐μm cell strainer, and washed with PBS. The spheroids/organoids that stayed in the strainer were collected and cultivated in H14 medium supplemented with 2% FBS. Routinely, after one‐night cultivation, cultures were rewashed and resuspended in fresh medium, and then used for further experiments.

### 2.4 Primary monocytes

Human peripheral blood mononuclear cells (PBMC) were isolated from buffy coats from healthy blood donors (The Blood Bank, Oslo University Hospital (Ullevaal), Norway) using Ficoll‐Hypaque density gradient centrifugation and following the standard protocol. PBMC (1.5 × 10^6^ cells·cm^−2^) were seeded in serum‐free X‐Vivo 15 medium (Lonza, Basel, Switzerland) supplemented with 2 mm GlutaMAX and antibiotics. Primary monocytes were enriched using plastic adherence before using them for further experiments.

### 2.5 Recombinant proteins and drugs

Human recombinant protein S100A4 (rS100A4) was produced as described previously (Berge *et al*., 2011) and used at 2 μg·mL^−1^. The protein stock had no detectable endotoxins as assayed by Lonza Biosciences. The specificity of rS100A4 to induce cytokines has been validated previously using of a S100A4‐blocking antibody (Bettum *et al*., 2014). Granulocyte–macrophage colony‐stimulating factor (GM‐CSF) and macrophage colony‐stimulating factor (M‐CSF) were purchased from R&D Systems (Oxon, UK). Carboplatin and paclitaxel were from Hospira Nordic (Sweden) and Fresenius Kabi (Norway), respectively.

### 2.6 Preparation of conditioned medium (CM) from BCCs

BCCs (40 000 cells·cm^−2^) were cultured in their respective medium overnight, washed, and incubated in 0.08 mL·cm^−2^ serum‐free DMEM/F12 supplemented with GlutaMAX, HEPES, and antibiotics. Subsequently, 2 μg·mL^−1^ rS100A4 or the equivalent amount of PBS was added, and 24 h later, CM‐S100A4 and CM‐Ctr, respectively, were collected. To prepare CM depleted for rS100A4, the BCCs were prestimulated with rS100A4 (or PBS) for 24 h, washed, and received the fresh medium, and after 24 h, CM‐S100A4‐0 and the respective CM‐Ctr‐0 were collected. All CMs were spun to remove cell debris before analysis of cytokine levels or further use on monocytes. For the latter, CMs were up‐concentrated using Amicon Ultra tubes with 3‐kDa MW cutoff (Millipore, Billerica, MA, USA) and diluted in fresh medium to 50% of the original volume to obtain twofold up‐concentration.

### 2.7 Treatment of monocytes with CMs

Human primary monocytes were cultured in CMs or ordinary medium (serum‐free X‐Vivo 15) with/without control additives (rS100A4, M‐CSF, or GM‐CSF) for 7 days. Cultures were observed by microscopy to evaluate morphological changes (images were obtained using an Olympus IX81 inverted microscope and the Olympus cellp imaging software, Tokyo, Japan). Cells harvested by EDTA and gentle scraping were analyzed for the levels of polarization markers by flow cytometry.

THP1 monocytes (25 000 cells·cm^−2^) were cultured in CMs or ordinary medium RPMI (all supplemented with 10% FBS) with/without rS100A4 for 7 days. Immature, unattached viable (i.e., trypan blue‐negative) monocytes were counted by a Countess automatic cell counter (Invitrogen, Carlsbad, CA, USA). The harvested cells were used for analysis of gene expression.

For analysis of cytokines, the THP1 cells were cultured in CM‐S100A4 or CM‐Ctr for 7 days and washed, and the same number of cells from both conditions were cultured further in serum‐free ordinary medium (0.08 mL·cm^−2^) for 3 days, before their growth medium was analyzed.

### 2.8 Effects of THP1 on BCCs in cocultures

To prepare TAM‐like THP1 and Ctr THP1, the THP1 monocytes (38 000 cells·cm^−2^) were cultured for 3 days in CM‐S100A4 or CM‐Ctr from MDA468, respectively. The resulting THP1 were collected by the help of TrypLE Express reagent (Gibco) and put in coculture with naïve MDA468 cells labeled with GFP‐Luc (cell ratio 1 : 1, DMEM/4% FBS medium).

The cocultures were grown for 6 days before they were analyzed for epithelial–mesenchymal transition (EMT) markers: intracellular localization of E‐cadherin (by immunofluorescence) or gene expression. For the latter, the cocultured cells were harvested by trypsination, THP1 cells were depleted using anti‐CD45 immunomagnetic beads (Dynabeads^®^#111.53, Dynal, Norway), and the remaining MDA468 cells were subjected to RNA isolation and further analysis by real‐time PCR.

To analyze MDA468 cell morphology/eccentricity and confluence, the GFP^+^ cancer cells in the cocultures were tracked by the IncuCyte ZOOM™ live cell imaging system (Essen Bioscience, Hertfordshire, UK). To assess MDA468 cell proliferation, the Luc‐mediated bioluminescence was measured as described previously (Seip *et al*., 2016). Briefly, after the addition of 0.1 mg·mL^−1^ D‐luciferin (Biosynth AG, Staad, Switzerland), bioluminescence was recorded 10 min later by a plate reader, Victor X3 (Perkin Elmer, Waltham, MA, USA).

To evaluate MDA468 cell sensitivity to chemotherapy, the cocultures (prepared in Corning^®^ Costar^®^ 96‐well white plates) were treated for 3 days, and the Luc^+^ cancer cell proliferation/viability was scored by measuring bioluminescence as described above.

To asses MDA468 cell migration, a tumor spheroid‐based assay described previously (Vinci *et al*., 2013) was used. Briefly, GFP^+^ MDA468 cells (4000/96‐well) were allowed to self‐assemble into spheroids in the presence of 2.5% Matrigel (BD Biosciences) in Corning^®^ Costar^®^ round‐bottomed ultralow attachment plates. Single spheroids were transferred into flat‐bottomed wells that were preseeded with Ctr THP1 or TAM‐like THP1 (1500 cells/96‐well). The wells were analyzed using Olympus fluorescence microscope and the ‘arbitrary structure measurement’ function of the cellp imaging software. Migration was quantified as follows: the total area covered by GFP^+^ cells at day 3 minus area of the initial spheroid.

### 2.9 Multiplex analyses of cytokines

The collected CMs/growth medium were stabilized using 1% human serum albumin Albunorm (Octapharma AG, Lachen, Switzerland), stored at −80 °C, and analyzed for the cytokine levels using a Bio‐Plex Pro™ Human Cytokine 27‐plex Assay # M50‐0KCAF0Y (Bio‐Rad, Hercules, CA, USA), a Luminex 200™ instrument (Luminex Corporation, Austin, TX, USA), and the bio‐plex manager 6.0 software (Bio‐Rad). Cytokine concentrations were determined from standard curves of known concentrations of recombinant proteins.

Cluster heatmaps of cytokine levels were generated in r (version 3.2.2) using rstudio (version 1.0.136) and the R package Clustermap (Lingjærde, personal communication). In brief, cytokine data were log10‐transformed and median‐centered (only cytokines detected in at least half of the samples were included). Untreated control samples were clustered using Euclidean distance and complete linkage, and the resulting order of cell lines and cytokines was used for visualizing both control and S100A4‐stimulated samples in the heatmaps in Fig. 2A.

### 2.10 RNA isolation, real‐time qPCR, and TCGA data analysis

Total RNA was isolated using the TRI Reagent^®^ (Invitrogen), and 1 μg RNA was reverse‐transcribed using qScript cDNA Synthesis Kit (Quanta Biosciences, Gaithersburg, MD, USA). Real‐time PCRs were performed on the iCycler instrument (Bio‐Rad). All reactions were run in parallel using 40 ng cDNA/sample, mixed with 200 nm FAM‐labeled probe [from the Universal ProbeLibrary collection (Roche Applied Science, Penzberg, Germany)], 250 nm of each primer, and 1x PerfeCTa qPCR Supermix (Quanta BioSciences). Primers were designed using the probe finder software from Roche Applied Science available online at the Universal ProbeLibrary Assay Design Center. Primer sequences/probe numbers are listed in Table S1. Relative gene expression was calculated by the ∆∆*C*_t_ method.

We used TCGA's BC cohort (Cancer Genome Atlas Network, 2012), *n* = 1052, to assess the expression pattern of genes of interest in different subtypes of BC. Gene expression was assayed by RNA sequencing, RSEM (RNAseq by Expectation‐Maximization; Li *et al*., 2010) normalized per gene. The data were obtained from TCGA's dbGAP data portal and log2‐transformed prior to analysis. Subtypes were called using PAM50 (Parker *et al*., 2009). We also used PAM50s value for evaluation of how correlated a tumor is to the basal‐like subtype. Analysis was performed in r (version 3.3.3). The tumor cell percentage in each sample was estimated from the SNP array data using the algorithm ASCAT (Allele‐Specific Copy number Analysis of Tumors) (Van Loo *et al*., 2010). Abundance of stroma and immune cells in the samples was inferred from gene expression signatures by ESTIMATE (Estimation of STromal and Immune cells in MAlignant Tumors using Expression data; Yoshihara *et al*., 2013). The abundance of different subsets of immune cells, including macrophages, was evaluated based on gene expression data using TIMER (Tumor Immune Estimation Resource; Li *et al*., 2017).

### 2.11 Immunohistochemistry (IHC) and immunofluorescence (IF)

For IHC, formalin‐fixed paraffin‐embedded tumor sections (3 μm) were pretreated in an automated PT‐link system (Dako, Glostrup, Denmark) before staining with 1 : 500 diluted rabbit anti‐S100A4 (Dako) for 30 min at room temperature followed by Dako Cytomation EnVision+ System‐HRP suitable for rabbit primary antibodies. The pictures were obtained using Olympus IX81 microscope and the cellp imaging software. For CD68 staining, the sections (after deparaffinization and heat‐induced epitope retrieval) were stained with 1 mg·mL^−1^ rabbit anti‐CD68 (Abcam, Cambridge, UK) for 1 h at room temperature followed by the MACH‐3 rabbit HRP‐polymer detection kit and the Betazoid DAB Chromogen kit (Biocare Medical, Concord, CA, USA). The stained sections were scanned using NanoZoomer HT digital slide scanner (Hamamatsu Photonics, Hamamatsu, Japan)

For IF, the cocultures were fixed in 4% paraformaldehyde, permeabilized with 0.5% Triton X‐100, and stained with 1 : 200 diluted (in PBS/0.05% saponin) mouse anti‐E‐cadherin antibody (Cell Signaling, Danvers, MA, USA) overnight at 4 °C. After staining with donkey anti‐mouse Alexa 555 (Jackson ImmunoResearch, West Grove, PA, USA, diluted 1 : 500) for 1 h at room temperature, the samples were mounted in ProLong Gold mounting medium containing DAPI (Life Technologies, Carlsbad, CA, USA). Fluorescent images were obtained using Zeiss LSM710 confocal microscope (Zeiss, Oberkochen, Germany) equipped with Plan‐Apochromat X 63/1.4 Oil DICII objective and analyzed using the zen 2011 software.

### 2.12 Flow cytometry

Cell staining was performed in a staining buffer (PBS/1 mg·mL^−1^ hu γ‐Globulins (Sigma), aggregated at 63 °C for 20 min) for 30 min at 4 °C with anti‐CD206‐PE and anti‐CD80‐PE (BD Biosciences). The samples were analyzed on a BD LSRII flow cytometer controlled by bd facsdiva software (San Jose, CA, USA). Dead cells were excluded from the analyses by prestaining with propidium iodide (Sigma). flowjo software (FlowJo, Ashland, OR, USA) was used for the data analysis.

For separation of epithelial cells from the remaining cells in the primary cultures, the cells were stained with anti‐EpCAM‐APC (Biolegend, San Diego, CA, USA) and sorted into EpCAM‐positive and EpCAM‐negative subpopulations by BD FACSDiva flow cytometer.

### 2.13 Statistical analysis

Statistical analysis was performed using two‐tailed Student's *t*‐test or the nonparametric Kruskal–Wallis test. For TCGA data analysis, we used *t*‐test to assess significant difference in the boxplot with two variables, and Kruskal–Wallis rank‐sum test to assess significant difference in the boxplot with more than two variables. Differences were considered statistically significant if *P*‐values were below 0.05.

## 3 Results

### 3.1 S100A4 levels correlate with abundance of stroma and immune cells in BC tissue

**Figure 1:**
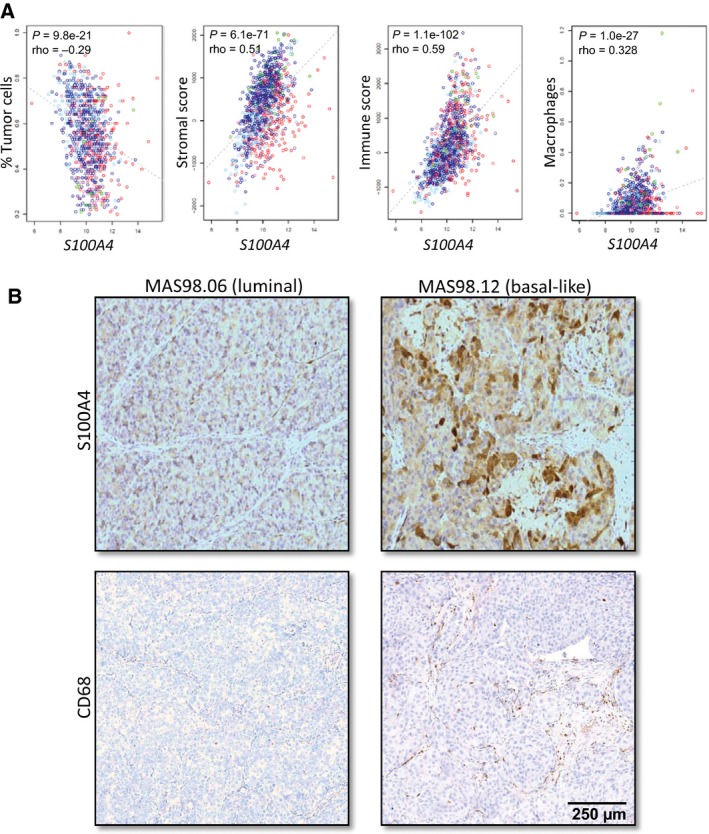
Association between S100A4 expression and abundance of stroma and immune cells in BC tissue. (A) In silico methods (specified in Materials and methods) were applied to estimate the percentage of tumor cells, stromal score, immune score, and macrophage abundance in the tumor samples from TCGA's breast cancer cohort (*n* = 1052). The correlation between the estimated parameters and S100A4 transcript levels is indicated (the colors specify BC subtypes: red: basal‐like; dark and light blue: luminal A and B, respectively; violet: HER2; and green: normal‐like). (B) The levels of S100A4 and infiltrated macrophages in the PDX models, MAS98.06 (luminal) and MAS98.12 (basal‐like). The representative images show IHC staining of S100A4 protein and the macrophage‐specific marker CD68. Scale bar: 250 μm for all the images.

We used *in silico* methods to evaluate the percentage of tumor cells and abundance of stroma (stromal score) and immune cell infiltration (immune score) in tumor samples from TCGA's BC cohort (*n* = 1052). Further, we evaluated how these parameters correlated with S100A4 expression. We observed an inverse correlation between S100A4 transcript levels and the percentage of tumor cells (Fig. 1A). In contrast, S100A4 transcript levels correlated positively with abundance of both stroma and immune cells, which was true for different subsets of immune cells: T cells, dendritic cells, neutrophils (data not shown), and macrophages (Fig. 1A). Also in the PDX models, we observed that MAS98.12 tumors with high levels of S100A4 were more infiltrated with macrophages (identified by CD68 staining) than MAS98.06 tumors with low S100A4 levels (Fig. 1B). Taken together, this suggests that in BC, S100A4 is primarily associated with the stromal component, and as such, could act extrinsically on malignant cells modulating their immune interactions.

### 3.2 Extracellular S100A4 stimulates breast cancer cells to enhance production of pro‐inflammatory cytokines

**Figure 2:**
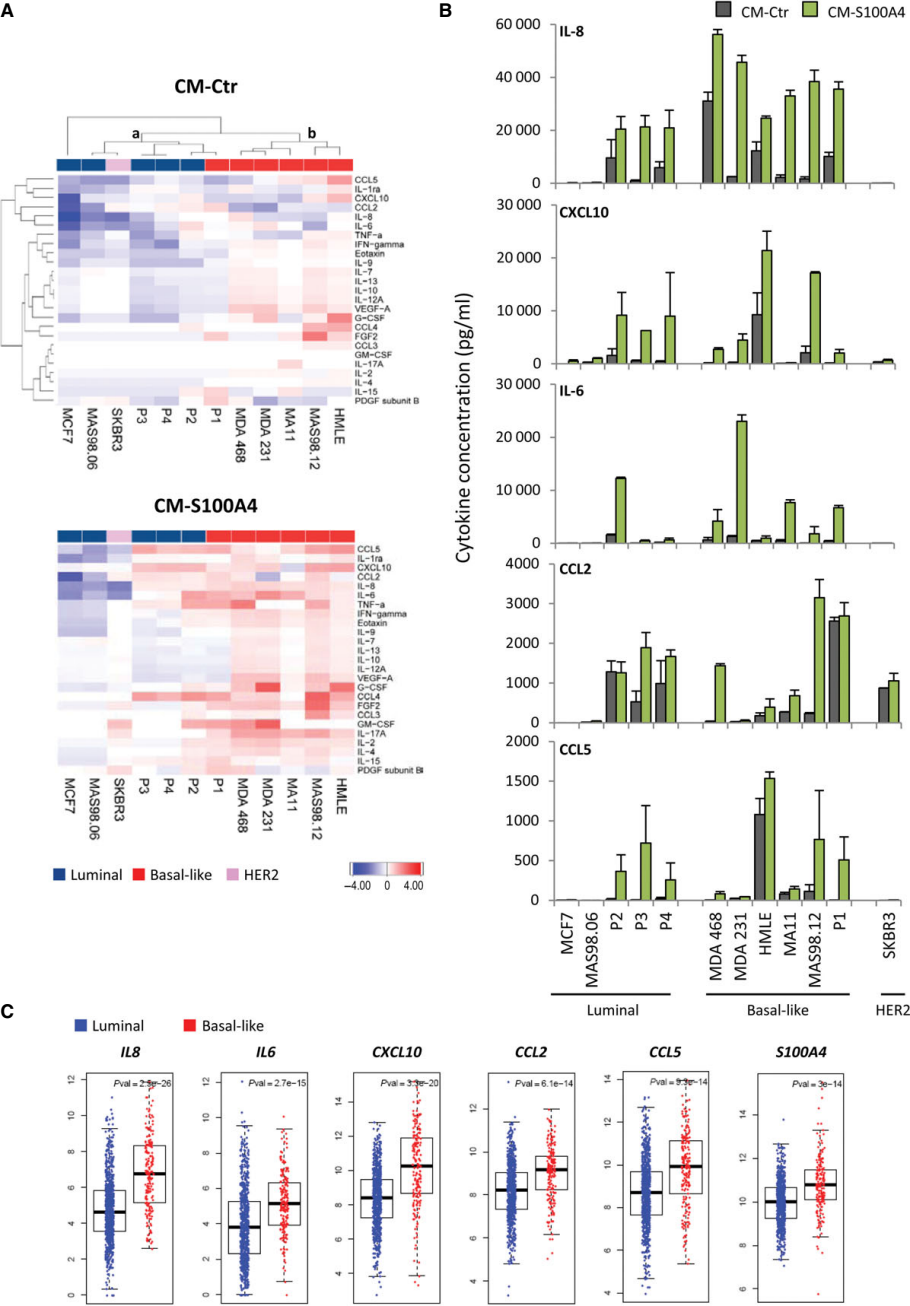
Cytokines secreted by BCCs at control and S100A4‐stimulated conditions. Cytokine levels were assessed in a panel of BCCs, which include cell lines (MCF7, MDA468, MDA231, HMLE, MA11, SKBR3), *ex vivo* cultures from PDXs (MAS98.06 and MAS98.12), and primary cultures from patient biopsies (P1, P2, P3, and P4) that represented different BC subtypes as indicated. CMs were harvested from nonstimulated controls (CM‐Ctr) and cells stimulated with 2 μg·mL^−1^ rS100A4 for 24 h (CM‐S100A4), and analyzed for cytokines by the multiplex immunoassay. (A) Heatmaps of 25 of 27 cytokines (IL‐1β and IL‐5 were excluded as their levels were zero in the majority of the samples) across different BCCs in CM‐Ctr (top panel) and CM‐S100A4 (bottom panel). The cytokine data (average from two independent experiments) were log10‐transformed and median‐centered, CM‐Ctr data were clustered, and the resulting order of the BCCs and cytokines was used for visualizing both CM‐Ctr and CM‐S100A4 data. Two clusters (a and b) are shown. (B) Average concentrations (pg·mL^−1^) of five most abundant cytokines secreted by the individual BCC models in two independent experiments (error bars indicate SEM). (C) Gene expression levels of the most abundant S100A4‐inducible cytokines in luminal and basal‐like tumors (the data retrieved from TCGA's breast cancer cohort).

To assess BCCs in control conditions and upon stimulation with extracellular S100A4, we analyzed cells of different origins (cell lines, *ex vivo* cultures from PDXs, and primary cultures from patient biopsies) and representing different BC subtypes, with respect to their ability to secrete cytokines. Conditioned media from nonstimulated control cells (CM‐Ctr) and cells stimulated with rS100A4 (CM‐S100A4) were analyzed by multiplex immunoassay measuring levels of 27 cytokines. The data for 25 detected cytokines across different BCCs are presented as heatmaps in Fig. 2A. Hierarchical clustering of the cytokine data in the control conditions (Fig. 2A top panel) separated two main clusters – luminal BCCs (cluster a) and basal‐like BCCs (cluster b) – where the later were enriched for most of the cytokines. A similar pattern was observed also in S100A4‐stimulated conditions (Fig. 2A bottom panel). Comparison of the cytokine profiles in CM‐S100A4 *versus* CM‐Ctr revealed a clear cytokine induction upon S100A4 stimulation, especially in basal‐like samples (Fig. 2A). Figure 2B specifies the concentrations of the five most abundant cytokines: IL‐8 (the dominant cytokine), IL‐6, CXCL10, CCL2, and CCL5, indicating their enrichment in CM‐S100A4, although a model‐to‐model variation should be noted. To validate that in the heterogeneous primary cultures, cytokine induction was due to response of the cancer cells and not the stromal cells, we separated cells positive for the epithelial marker EpCAM from the EpCAM‐negative counterparts. Only the EpCAM‐positive cell subpopulations consisting primarily of BCCs responded to S100A4 by increasing cytokine production (Fig. S1). Taken together, this indicates that basal‐like BCCs, especially when stimulated with S100A4, create a microenvironment strongly enriched for numerous cytokines, particularly IL‐8, IL‐6, CXCL10, CCL2, and CCL5, known ‘messengers’ of immune interactions. Interestingly, these five cytokines, as well as S100A4 itself, were found to be upregulated at the mRNA level in basal‐like tumors compared to tumors of the luminal subtype (Fig. 2C and Fig. S2). To note, the basal‐like PDX MAS98.12 also had high levels of S100A4 and, interestingly, were more infiltrated with macrophages than the luminal PDX MAS98.06 (Fig. 1B).

### 3.3 S100A4‐activated basal‐like BCCs trigger monocyte‐to‐macrophage differentiation and polarization

**Figure 3:**
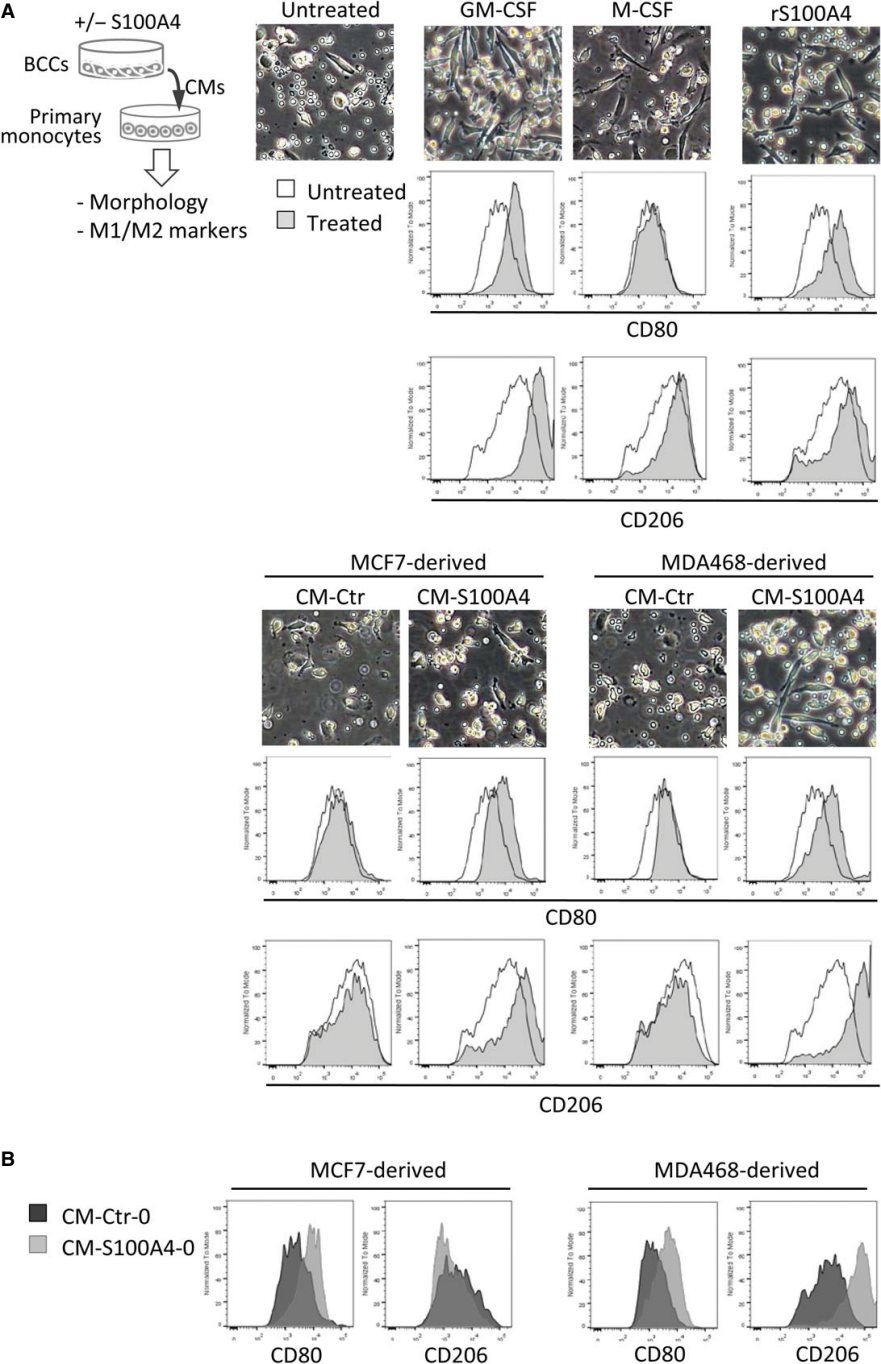
Effect of BCC‐derived CMs on primary human monocytes. (A) The experimental setup is depicted in the cartoon. Primary human monocytes were cultivated for 7 days in the presence of indicated factors: GM‐CSF or M‐CSF (both 50 ng·mL^−1^), rS100A4 (2 μg·mL^−1^), CM‐Ctr, and CM‐S100A4 from MCF7 or MDA468 BCCs. Changes in monocyte morphology and expression of M1 and M2 polarization markers, CD80 and CD206, were analyzed by microscopy and flow cytometry, respectively. Representative phase‐contrast images from each condition are shown in the upper panel. Histogram overlays indicate the levels of CD80 and CD206 under each treatment condition (filled histograms) compared to the untreated control (unfilled histograms). (B) Levels of CD80 and CD206 in primary monocytes cultured in CM‐S100A4‐0 (depleted for rS100A4) *versus* the respective control CM‐Ctr‐0 from MCF7 and MDA468.

Given that S100A4‐induced cytokines can act on myeloid cells, we further studied their influence on monocyte differentiation and polarization. Freshly isolated human monocytes were cultured in CM‐Ctr or CM‐S100A4 from luminal MCF7 or basal‐like MDA468 cells (poor and good responders to S100A4, respectively). The fate of the monocytes was analyzed by observing morphological changes, and by measuring the levels of CD80 and CD206 proteins, markers of M1 and M2 polarization, respectively. As shown in Fig. 3A, MDA468‐derived CM‐S100A4 supported monocyte survival and promoted elongated, macrophage‐like morphology more than the respective CM‐Ctr, or any CM from MCF7. Furthermore, the CM‐S100A4, but not the CM‐Ctr, notably increased the level of CD206, with less change in CD80. Changes in morphology and increase in CD206/CD80 levels were also observed after treatment with GM‐CSF and M‐CSF, known inducers of macrophage differentiation/polarization that we used to confirm the expected responsiveness in our monocytes. Of note, rS100A4 alone also promoted elongated morphology and upregulation of CD206/CD80 in the primary monocytes, but to a lower degree than the CM‐S100A4 from MDA468. Further, we employed CM‐S100A4‐0, which was depleted for the recombinant protein (see Materials and methods). CM‐S100A4‐0 from MDA468, but not MCF7, also induced upregulation of CD206 and CD80 by 13‐ and 3‐fold, respectively, compared to CM‐Ctr‐0 (Fig. 3B). This validates that factors secreted by S100A4‐activated BCCs affect primary human monocytes and that the basal‐like MDA468 has a stronger influence than the luminal MCF7.

**Figure 4:**
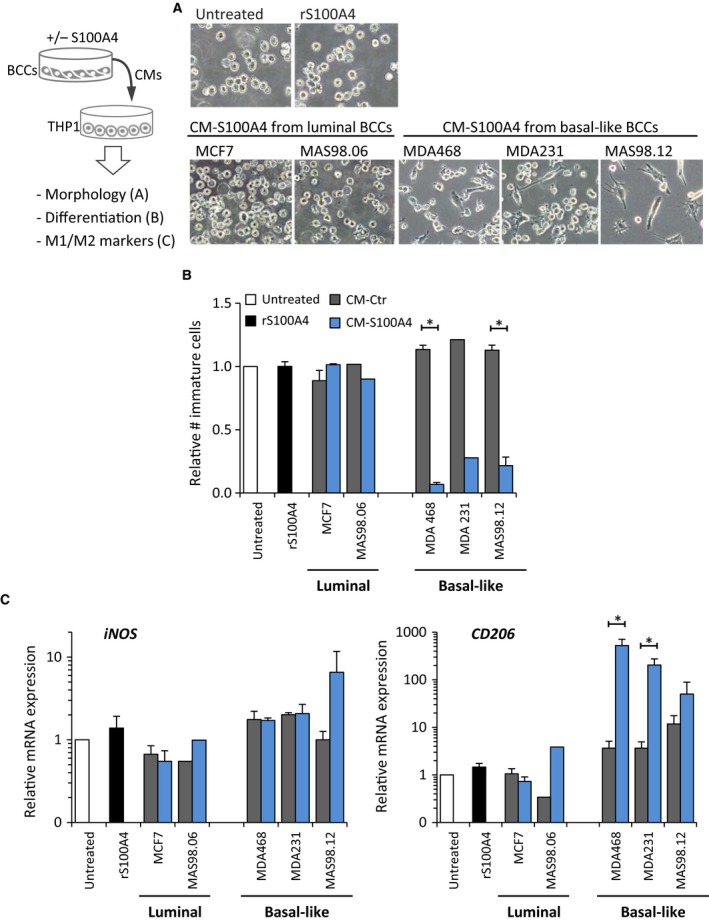
S100A4‐activated basal‐like BCCs stimulate THP1 monocyte differentiation and polarization toward M2 phenotype. The experimental setup is depicted in the cartoon. THP1 monocytes were cultured for 7 days in CM‐Ctr or CM‐S100A4 from luminal or basal‐like BCCs and analyzed for their morphological changes (A), number of immature cells that is a reverse measure of differentiation (B), and expression of genes reflecting M1 and M2 polarization (C). ‘rS100A4’ indicates treatment with 2 μg·mL^−1^ rS100A4 alone. (A) Representative phase‐contrast images of THP1 cells under indicated conditions; THP1 cultured in CM‐Ctr did not demonstrate any morphological changes compared to ‘Untreated’ THP1 and are therefore not shown. (B) Relative number of immature THP1 cells under each condition. (C) Relative mRNA expression of M1 and M2 polarization markers, iNOS and CD206, respectively, in THP1 cells under each condition compared to the untreated control (set to 1); average ± SEM (*n* = 3; no SEM indicates *n* = 1); *P < 0.05.

To investigate further whether the capacity to influence monocytes was linked to a BC subtype, we employed the monocytic cell line THP1. THP1 monocytes have been used previously to study BC subtype‐dependent effects (Stewart *et al*., 2012). Besides, THP1 monocytes show minimal response to rS100A4 alone (Fig. 4) and are an ideal model to study the effects of CM‐S100A4. THP1 monocytes were cultured in the presence of CM‐S100A4 or CM‐Ctr from two luminal and three basal‐like BCCs, representing both cell lines and *ex vivo* cultures from PDXs. The fate of THP1 cells was evaluated based on their morphology, number of immature unattached cells (THP1 adhere to the surface and stop proliferation upon differentiation), and expression of genes linked to M1/M2 polarization. As previously, we observed that CM‐S100A4 from the basal‐like BCCs induced an adherent, macrophage‐like morphology in THP1 cells (Fig. 4A). This effect was concomitant with significant reduction in the number of immature monocytes (Fig. 4B), which is an indication of macrophage differentiation. Furthermore, such THP1 cells significantly upregulated *CD206*, a marker of M2 polarization, but showed no major changes in expression of *iNOS*, a marker of M1 polarization (Fig. 4C). In contrast, CMs from the luminal‐like BCCs did not induce any of these changes in THP1 cells.

### 3.4 S100A4‐activated basal‐like BCCs educate macrophages to produce cytokines

**Figure 5:**
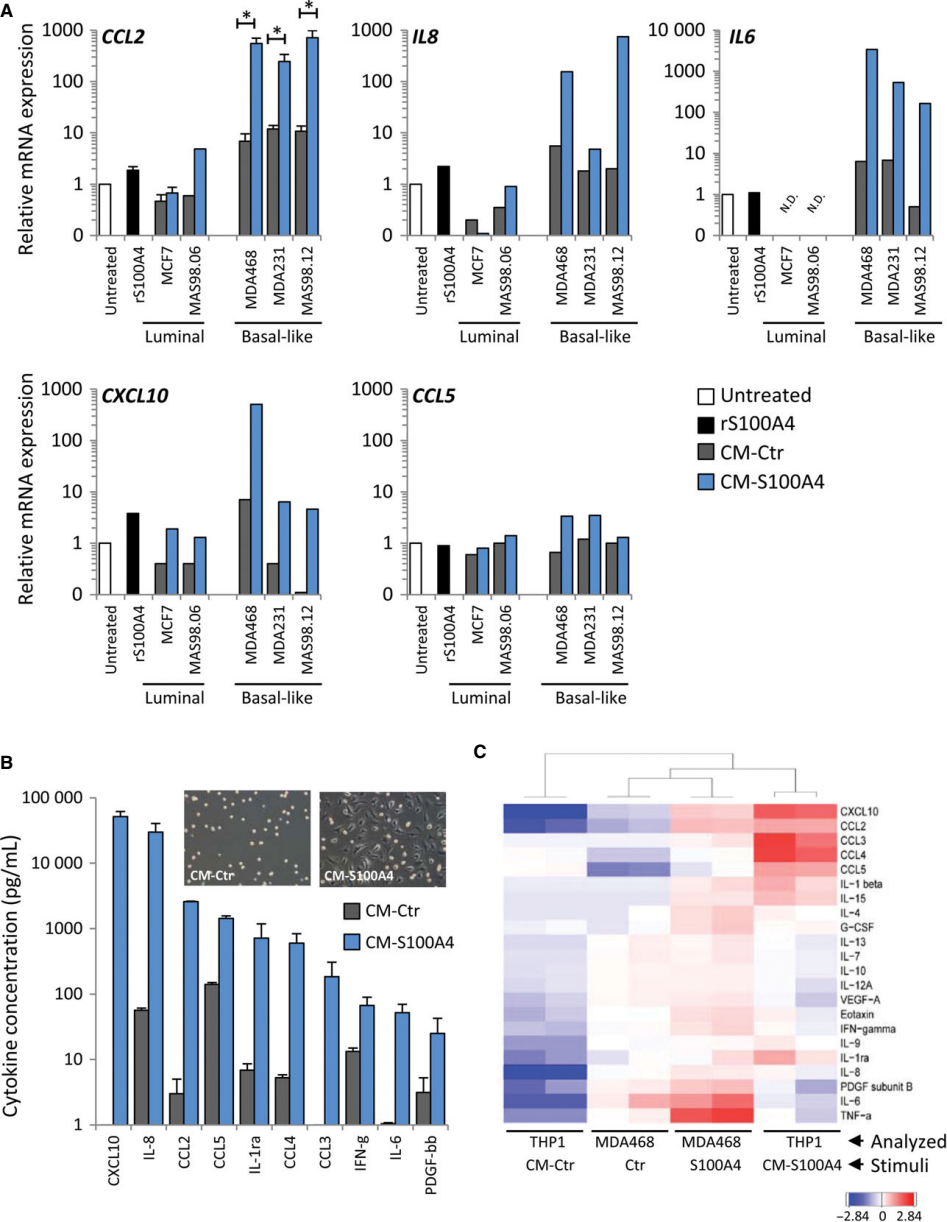
S100A4‐activated basal‐like BCCs educate THP1 cells to produce cytokines. (A) THP1 cells were cultured for 7 days in CM‐Ctr or CM‐S100A4 from luminal or basal‐like BCCs (‘rS100A4’ – treatment with 2 μg·mL^−1^ rS100A4 alone) and analyzed for expression of cytokine genes. Relative mRNA expression of indicated cytokines under each condition compared to untreated controls (set to 1). For CCL2, average ± SEM (*n* = 3, except MAS98.06, where *n* = 1); the other cytokines were measured in one representative sample from each BCC model. N.D. indicates below the detection limit. (B) THP1 cells were cultured for seven days in CM‐Ctr or CM‐S100A4 from MDA468, washed, and received an ordinary serum‐free medium, and after 3 days, their growth medium was analyzed for cytokines by multiplex immunoassay. The graph indicates the top 10 cytokines elevated in CM‐S100A4 compared to CM‐Ctr. Average concentrations (pg·mL^−1^) of cytokines from two independent experiments ± SEM. The morphology of the resulting THP1 cells is depicted in the insert. (C) Heatmap with hierarchical clustering of all detectable cytokines (log_10_‐transformed and median‐centered) in THP1 and MDA468 (Analyzed) in control and S100A4‐stimulated conditions (Stimuli). Two biological parallels for each condition are shown.

To characterize the BC‐educated THP1 cells further, we examined their ability to produce cytokines. THP1 monocytes were cultured in CM‐Ctr or CM‐S100A4 from the luminal and the basal‐like BCCs as previously, and analyzed for expression of the five cytokines highly produced by BCCs themselves. We found that CM‐S100A4 from the basal‐like BCCs made the THP1 cells upregulate *CCL2*,* IL6*,* CXCL10*, and *IL8* (the upregulation of *CCL5* was negligible; Fig. 5A).

To confirm the gene expression results, we measured the levels of secreted cytokines. THP1 monocytes were cultured in CM‐S100A4 or CM‐Ctr from MDA468 as before, washed, and received an ordinary medium, and three days later, their growth medium was analyzed by multiplex immunoassay. The morphology of THP1 cells from CM‐Ctr and CM‐S100A4 is shown in the insert of Fig. 5B, indicating that the former kept unaltered, monocytic appearance, while the latter gained flat, macrophage‐like morphology. Such CM‐S100A4‐educated, macrophage‐like THP1 cells secreted significantly higher amounts of all detectable cytokines (Fig. 5B). CXCL10, IL‐8, CCL2, and CCL5 were among the top five most abundant cytokines, similar to what was observed in S100A4‐activated BCCs. To compare the cytokine profiles in educated THP1 and educating MDA468, we performed hierarchical clustering analysis. As shown in Fig. 5C, THP1 cultured in CM‐S100A4 clustered close to S100A4‐activated MDA468. This suggests that the CM‐S100A4‐educated THP1 macrophages produce cytokines that were involved in their education.

### 3.5 BC‐educated macrophages promote EMT, proliferation, chemoresistance, and migration in BCCs

**Figure 6:**
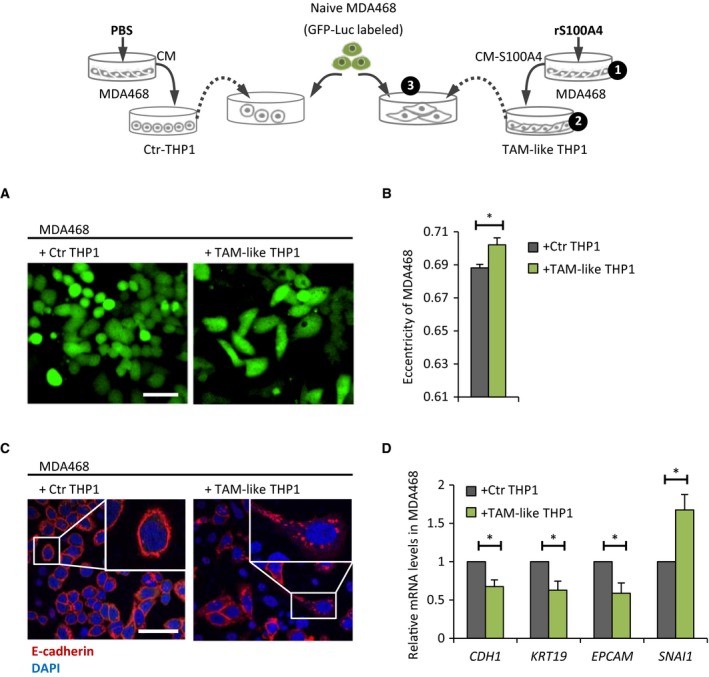
TAM‐like THP1 make MDA468 cells gain mesenchymal traits. The experimental setup is illustrated in the cartoon. Briefly, MDA468‐derived CM‐Ctr and CM‐S100A4 were prepared as before (step 1) and added to THP1 cells for 3‐day treatment to generate Ctr THP1 and TAM‐like THP1, respectively (step 2), which then were put in coculture together with naïve MDA468 cells labeled with GFP‐Luc for tracking purposes (step 3). The MDA468 cells from both cocultures were analyzed as follows: (A, B) Morphological changes were tracked by IncuCyte ZOOM ^®^ (A) and quantified at day 3 by measuring eccentricity (B); average ± SEM (*n* = 3). Scale bar: 50 μm. (C) Cellular localization of E‐cadherin (red) at day 6 was detected by IF [DAPI nuclear staining (blue)]. Scale bar: 50 μm. (D) Relative mRNA expression (at day 6) of indicated genes in the presence of TAM‐like THP1 *versus* Ctr THP1 (set to 1); average ± SEM (*n* = 3); *P < 0.05.

The effect of S100A4‐activated BCCs on macrophage differentiation, M2 polarization, and production of tumor‐promoting cytokines suggests the formation of protumorigenic TAM‐like cells. To demonstrate that CM‐S100A4‐educated macrophages act as TAMs and support BCCs, we used a three‐step model depicted in Fig. 6. Briefly, THP1 cells were cultured in CM‐Ctr (denoted as Ctr THP1) or CM‐S100A4 (further denoted as TAM‐like THP1) and put in cocultures with naïve MDA468 cells labeled with GFP‐Luc. The phenotypic characteristics and functional properties (proliferation, chemosensitivity and migration) of MDA468 cells in both cocultures were compared as described below.

We observed that in the presence of TAM‐like THP1, the MDA468 cells gained phenotypic alterations that resembled EMT. First, their morphology appeared elongated, mesenchymal‐like (Fig. 6A), which was measured as enhanced eccentricity (Fig. 6B). Second, E‐cadherin, a hallmark of epithelial phenotype, shifted its characteristic localization in the cell membrane to the intracellular space (Fig. 6C). Third, gene expression analysis revealed downregulation of epithelial markers (*CDH1*,* KRT19*, and *EPCAM*) and upregulation of the mesenchymal marker *SNAI1* (Fig. 6D).

**Figure 7:**
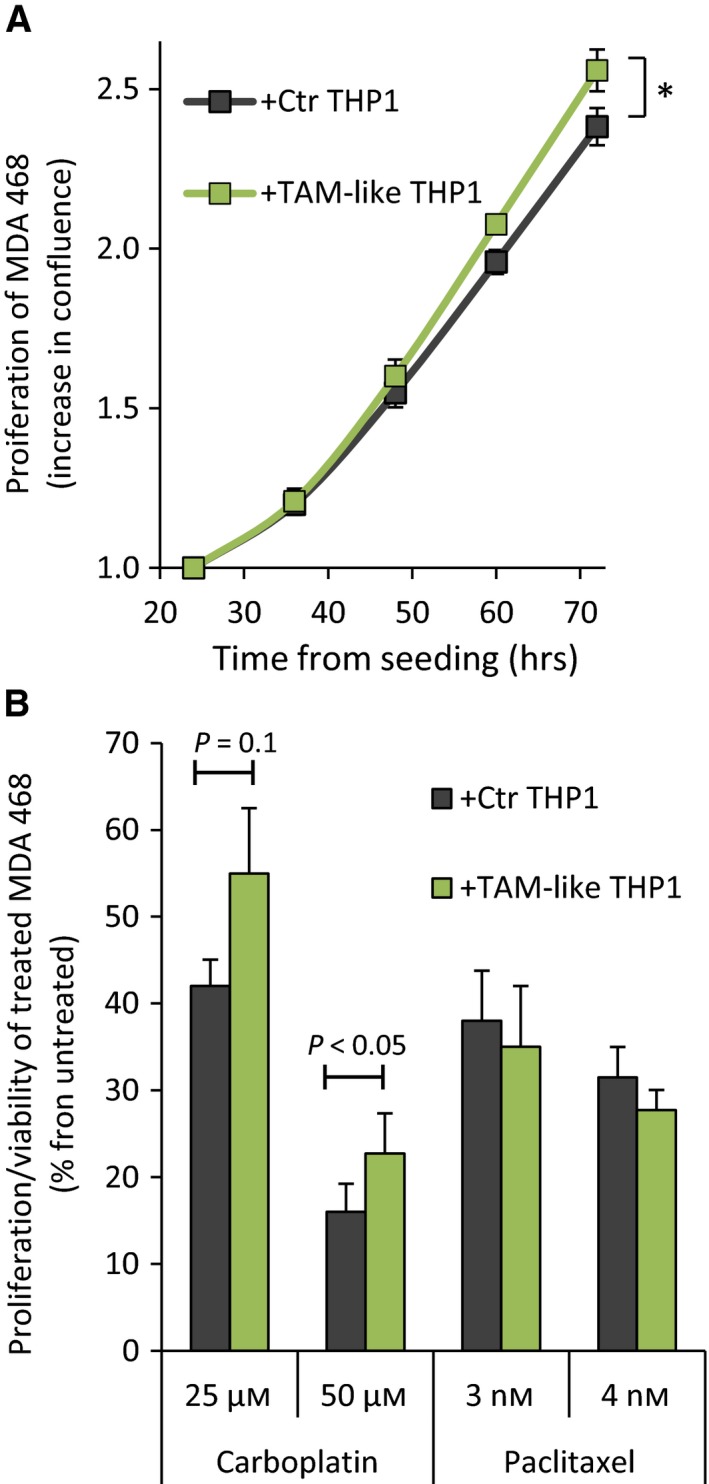
TAM‐like THP1 stimulate proliferation and reduce chemosensitivity of MDA468 cells. The cocultures of GFP‐Luc‐labeled MDA468 cells and Ctr THP1 or TAM‐like THP1 were prepared as illustrated in Fig. 6. (A) Cancer cell confluence/proliferation was tracked over time by IncuCyte ZOOM, recording the GFP signal. The confluence at 24 h postseeding was set to 1, and the increase in the confluence over time is indicated; average ± SEM (*n* = 5); *P < 0.05 for 48–72 h. (B) Both cocultures were treated with carboplatin or paclitaxel at the indicated concentrations for 3 days. The proliferation/viability of the treated cancer cells was scored by measuring Luc‐mediated bioluminescence and is presented as % from respective untreated controls (average ± SEM,* n* = 3–4).

To examine MDA468 cell proliferation, we tracked GFP^+^ cancer cell confluence over time in both cocultures. We observed a higher increase in confluence in the cocultures with TAM‐like THP1 than Ctr THP1 (Fig. 7A). In line with that, we detected 13 ± 5% difference (*P* = 10^−4^, *n* = 5) in bioluminescence, indicating higher proliferation of Luc^+^ MDA468 cells in the cocultures with TAM‐like THP1 (data not shown).

To investigate MDA468 cell sensitivity to chemotherapy, both cocultures were treated with either carboplatin or paclitaxel for 3 days. The proliferation/viability of the treated cancer cells was scored by measuring Luc‐mediated bioluminescence. We observed a lower antiproliferative effect of carboplatin (but not paclitaxel) in the MDA468 cells cocultured with TAM‐like THP1 compared to Ctr THP1 (Fig. 7B). The difference was not big, from 1.3‐ to 1.4‐fold, but consistent in all experiments (Fig. S3).

**Figure 8:**
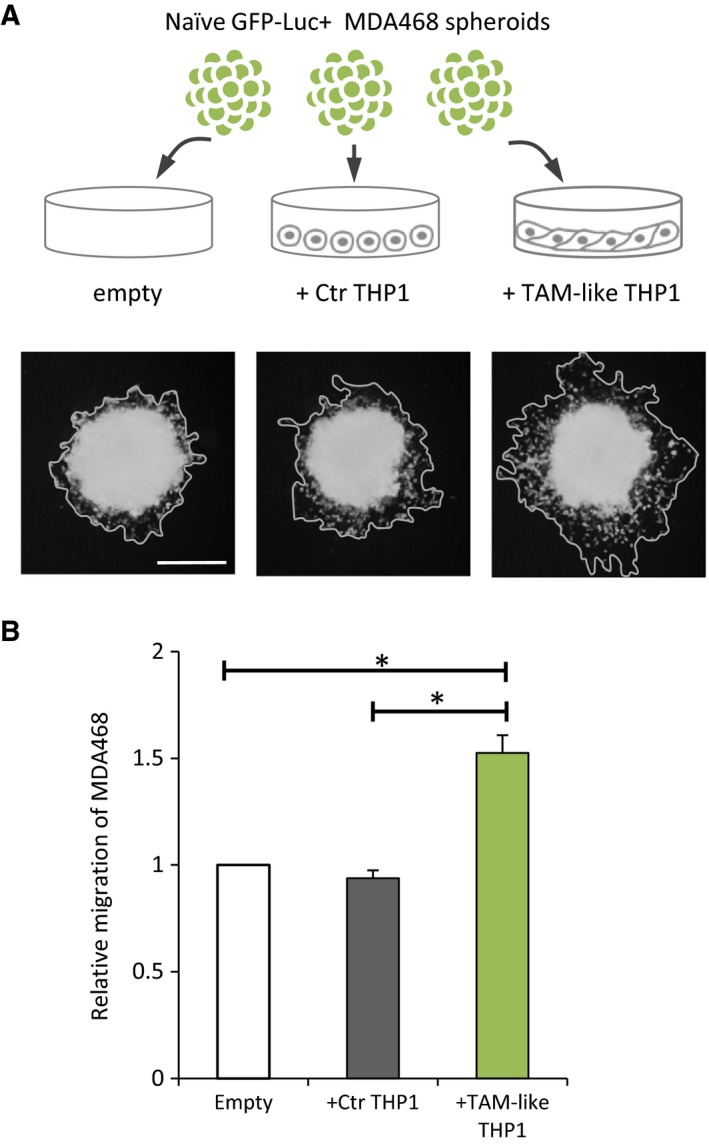
TAM‐like THP1 stimulate motility of MDA468 cells. (A) GFP‐Luc‐labeled MDA468 cells were formed into spheroids and cultured alone (empty) or together with Ctr THP1 or TAM‐like THP1 as illustrated. Representative microscopy pictures (at day 5) under each condition are shown, indicating the total area covered by the MDA468 cells. Scale bar: 500 μm for all the images. (B) Migration area, that is, total area minus initial spheroid area, was measured in each condition and normalized to the migration area in the ‘empty’ control (set to 1); average ± SEM (*n* = 3); *P < 0.05.

To examine MDA468 cell motility, the cancer cell spheroids were established and cultured alone or together with Ctr THP1 or TAM‐like THP1 as depicted in Fig. 8A. Based on the area covered by scattered MDA468 cells (total area minus initial spheroid area), TAM‐like THP1 promoted cancer cell migration by approximately 60% compared to Ctr THP1 (Fig. 8B).

**Figure 9:**
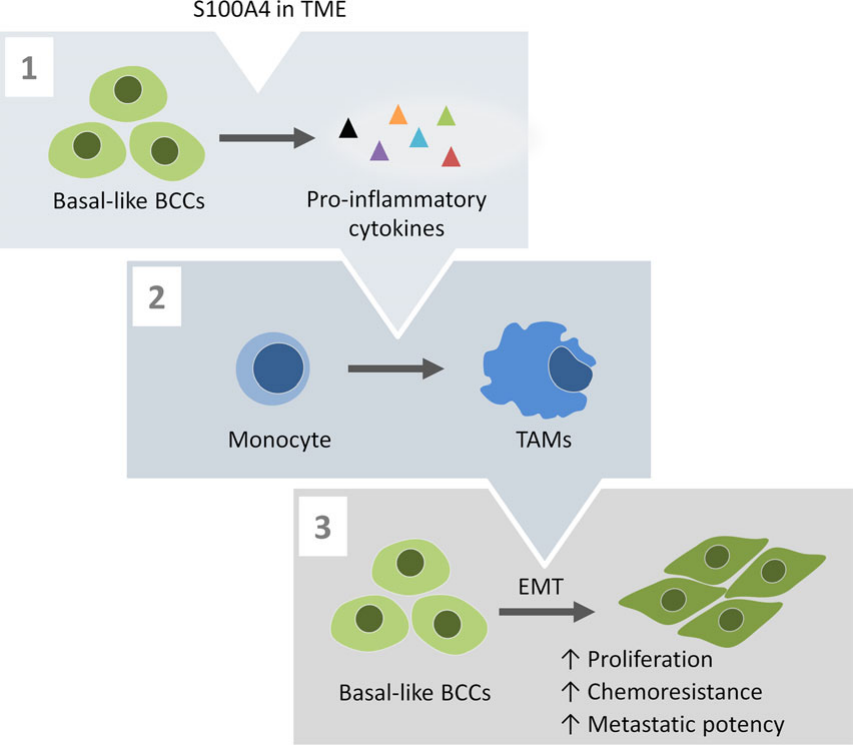
An illustrative summary of the findings. Basal‐like BCCs, when exposed to S100A4 present in TME, increase secretion of numerous pro‐inflammatory cytokines (1). The secreted factors convert monocytes into TAMs (2), which stimulate EMT and promote aggressive functions (proliferation, chemoresistance, and motility) in BCCs (3).

In summary, we have demonstrated that in the presence of extracellular S100A4, basal‐like BCCs, compared to luminal BCCs, present a richer cocktail of cytokines/secreted factors that educate monocytes into macrophages with TAM features (Fig. 9). Such educated TAM‐like cells equip cancer cells with more aggressive phenotypic/functional characteristics – mesenchymal traits, higher proliferation, higher chemoresistance, and enhanced motility – typical characteristics of basal‐like BC.

## 4 Discussion

Chronic inflammation is an important hallmark of cancer, and interactions between tumor cells and the inflammatory environment represent an attractive therapeutic target. In this study, we revealed a protumorigenic cascade of interactions between BCCs and myeloid cells, which was triggered by the microenvironmental factor S100A4. Such a mechanism is in line with observations from BC tumors, showing an association between elevated levels of S100A4 and abundance of both stroma and immune cells, including macrophages. This suggests that (a) S100A4 is primarily present in the TME and acts on BCCs as an extracellular factor and (b) S100A4 plays a role in recruiting immune/inflammatory cells to the tumors.

We documented that extracellular S100A4 stimulates BCCs to release a variety of pro‐inflammatory cytokines and other ‘messengers’ of immune interactions. Importantly, cancer cells from the aggressive basal‐like subtype were the most potent cytokine producers. Among the most abundant cytokines, we identified IL‐8, IL‐6, CXCL10, CCL2, and CCL5. This is in concordance with the observations in patient biopsies, where these cytokines were expressed at elevated levels in basal‐like tumors compared to the luminal tumors. As these cytokines are associated with poor prognosis and cancer progression (Benoy *et al*., 2004; Lv *et al*., 2013; Salgado *et al*., 2003), their abundance in basal‐like tumors may be linked to the aggressive behavior of this subtype. In the current study, we pursued this idea by studying the ability of BCC‐secreted factors to engage tumor‐supportive macrophages. We showed that upon S100A4 stimulation, basal‐like BCCs secrete factors that trigger monocyte‐to‐macrophage differentiation and polarization. This is in line with previous studies demonstrating that basal‐like/TNBC, compared to luminal BC, have superior abilities to recruit macrophages (Espinoza *et al*., 2016; Sousa *et al*., 2015), modulate their polarization, and stimulate protumorigenic functions (Hollmen *et al*., 2015; Sousa *et al*., 2015; Stewart *et al*., 2012; Su *et al*., 2014). The current study adds to the previous knowledge by revealing a strong potentiating influence of S100A4, which is expressed at elevated levels in basal‐like/TNBC as also reported previously (Egeland *et al*., 2017). Furthermore, TNBC shows elevated expression of TLR4 and RAGE receptors (Mehmeti *et al*., 2015; Nasser *et al*., 2015), which have been linked to S100A4‐trigged cytokine induction (Cerezo *et al*., 2014; Haase‐Kohn *et al*., 2011).

It should be mentioned that we have not pinpointed specific factors released by S100A4‐activated BCCs that were responsible for the effects on myeloid cells. IL‐4, IL‐10, and IL‐13 – the known inducers of M2 macrophages – are unlikely to be implicated as their levels were low (not exceeding 7, 70, and 120 pg·mL^−1^, respectively) despite S100A4 stimulation. The levels of GM‐CSF, found by Su *et al*. (2014) to mediate BCCs effects on macrophages, were also negligible in the majority of the examined BCC models, although 42 pg·mL^−1^ was detected in S100A4‐activated MDA468. This concentration, however, was lower than the concentration used to induce M2 macrophages in Su's study (Su *et al*., 2014). CCL2 and CCL5, on the other hand, were present in abundance and could mediate the effects on myeloid cells. These cytokines are primarily linked to monocyte recruitment and are known to be able to elevate TAM levels in tumors (Soria and Ben‐Baruch, 2008). Their role in modulating macrophage phenotype/functions is less clarified, although it has been demonstrated that CCL5 can promote prometastatic phenotype of TAMs (Frankenberger *et al*., 2015).

The ability of S100A4‐activated basal‐like BCCs to promote tumor‐supportive phenotype in macrophages has been verified by functional characterization of CM‐S100A4‐educated THP1, denoted TAM‐like THP1. Thus, we demonstrated that such TAM‐like THP1 produced elevated levels of protumorigenic cytokines, including the mentioned IL‐8, IL‐6, CXCL10, CCL2, and CCL5. Thereby, BC‐educated macrophages could function as amplifiers of cytokine production. This observation is in concordance with previous reports from TNBC, where it was shown that tumor‐infiltrating immune cells contribute to the total pool of tumor cytokines (Espinoza *et al*., 2016). Interestingly, Picon‐Ruiz *et al*. (2016) identified the same five cytokines to be upregulated in BC‐educated adipocytes and showed their protumorigenic influence. Next, we demonstrated that TAM‐like THP1 cells promoted a more aggressive phenotype in BCCs. The morphological, molecular, and functional alterations observed in cancer cells suggest induction of EMT. The most prominent effect was on their migratory capacity, proposing a potentiating role of the S100A4–BCC–TAM cascade in metastasis. Su *et al*. (2014) also reported acquisition of EMT traits and enhanced metastasis upon interaction with BC‐educated macrophages. We have also observed reduced sensitivity to the chemotherapeutic agent carboplatin in the presence of TAM‐like THP1. The potentiated chemoresistance might be associated with the induced EMT. Recently, we have documented such an association in malignant melanoma, which, upon interaction with stromal cells, switched to the mesenchymal phenotype and, simultaneously, became resistant to therapy (Seip *et al*., 2016). There could also be other, EMT‐independent mechanisms causing TAM‐like cells to facilitate chemoresistance. For example, TAM‐produced cytokines have been shown to stimulate survival signaling pathways in cancer cells and thereby attenuate sensitivity to therapy (Yang *et al*., 2015).

Here, we focused on myeloid cells as interaction partners of S100A4‐activated BCCs, but other immune cells can possibly be engaged through a similar S100A4‐triggered cascade. We did observe a correlation between S100A4 levels and infiltration of not only macrophages, but also other immune cells, *for example*, T cells. This is in line with Grum‐Schwensen *et al*. (2010), who demonstrated that extracellular S100A4 was involved in recruiting T lymphocytes and making them release cytokines, which, altogether, stimulated metastasis. Thus, S100A4‐rich tumors, like basal‐like BC, might possess advantageous mechanisms for engaging immune/inflammatory cells with protumorigenic activity.

## 5 Conclusions

We have shown that the prometastatic microenvironmental factor S100A4 stimulates basal‐like BCCs to secrete factors/cytokines that convert monocytes into TAM‐like cells demonstrating tumor‐supporting functions. The S100A4–BCC–TAM interaction cascade can potentiate both metastatic abilities and drug resistance and could be an important contributor to the aggressive behavior of basal‐like BC. Inhibition of such cascade should be explored for therapeutic intervention.

## Author contributions

LP, EMT, and GMM were involved in conception, design, and data interpretation. LP and EMT wrote the manuscript. EMT, SP, SJ, and AP were involved in data acquisition. LP, EMT, EVE, SN, and MHH were involved in data processing and graphical presentation. OE represented a link to the clinic and contributed to the data interpretation from a clinical perspective. VNK, ALBD, and GMM provided administrative, technical, and financial support. GMM supervised the study. All authors read and approved the manuscript.

## Supporting information


**Fig. S1.** S100A4‐induced cytokine production is observed in EpCAM‐positive BCCs, but not in EpCAM‐negative stromal cells.
**Fig. S2.** Gene expression levels of the most abundant S100A4‐inducible cytokines and S100A4 itself in the five molecular subtypes of BC from TCGA's collection of breast tumors.
**Fig. S3.** TAM‐like THP1 make MDA468 cells less sensitive to carboplatin.
**Table S1.** Primer sequences and probes used for qPCR.
