# Supplementary material for: Basal‐like breast cancer engages tumor‐supportive macrophages via secreted factors induced by extracellular S100A4
Source: Mol Oncol. 2018 Aug 9;12(9):1540–58. doi: 10.1002/1878-0261.12319 (PMC6120223; doi:10.1002/1878-0261.12319)
Supplement: Supplementary file 1 — Fig. S1. S100A4‐induced cytokine production is observed in EpCAM‐positive BCCs, but not in EpCAM‐negative stromal cells. Fig. S2. Gene expression levels of the most abundant S100A4‐inducible cytokines and S100A4 itself in the five molecular subtypes of BC from TCGA's collection of breast tumors. Fig. S3. TAM‐like THP1 make MDA468 cells less sensitive to carboplatin. Table S1. Primer sequences and probes used for qPCR. [file MOL2-12-1540-s001.pdf]

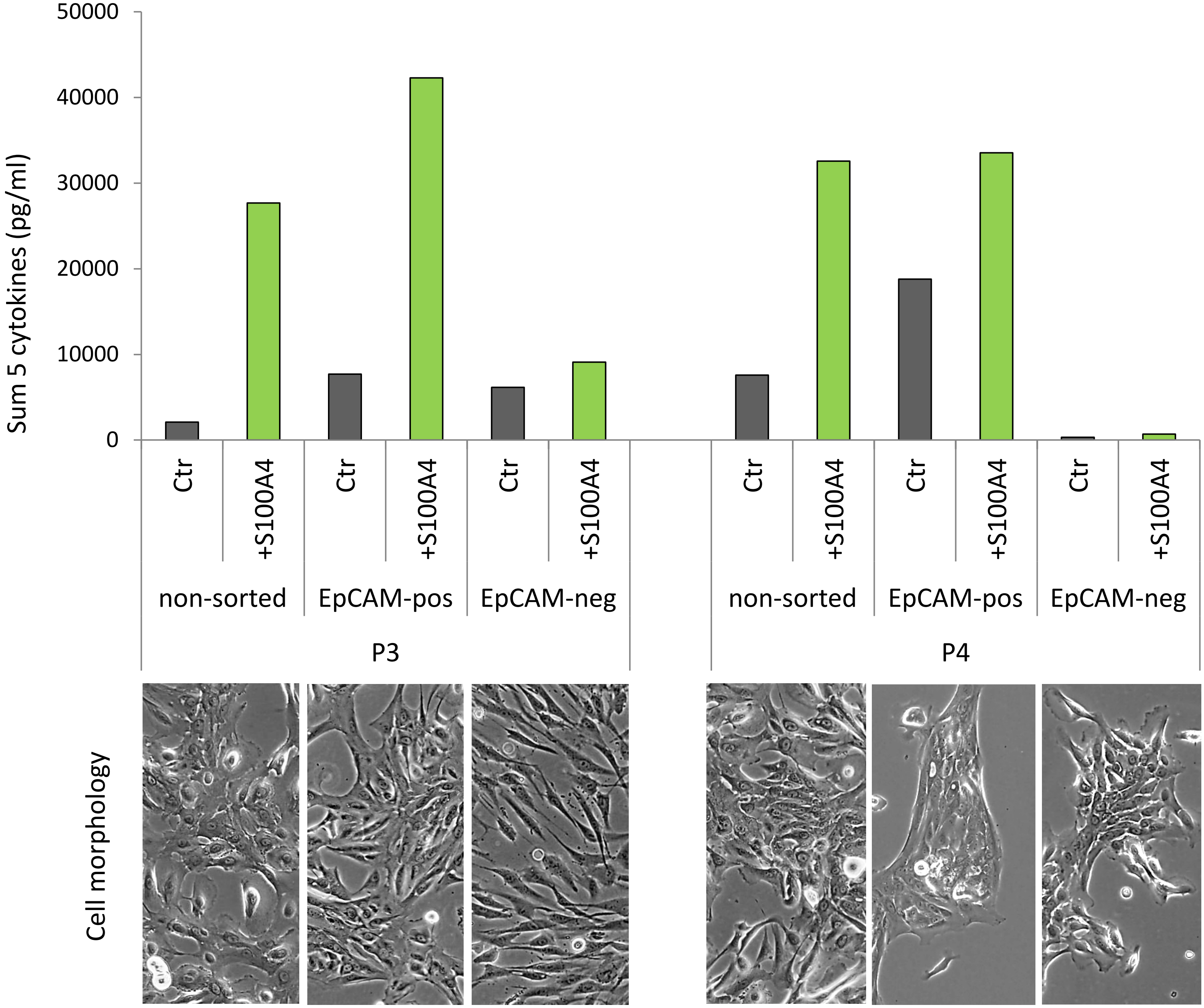

Supplementary Figure S1. **S100A4 induced cytokine production is observed in EpCAM positive BCCs, but not in EpCAM negative stromal cells.** Cells from two patient biopsies (P3 and P4) were propagated in culture and FACS sorted into EpCAM-positive (enriched for BCCs) and EpCAM-negative (enriched for stromal cells) subpopulations. Each subpopulation was stimulated with 2 μg/ml rS100A4 (controls, non-stimulated) for 24 hrs and the growth medium was analyzed for cytokine levels by the multiplex immunoassay. The total level of five most abundant cytokines (IL-8, IL-6, CXCL10, CCL2 and CCL5) in each subpopulation and non-sorted cells, with/without S100A4 stimulation, is presented in the upper graph. Phase contrast images illustrate the morphology of each culture.

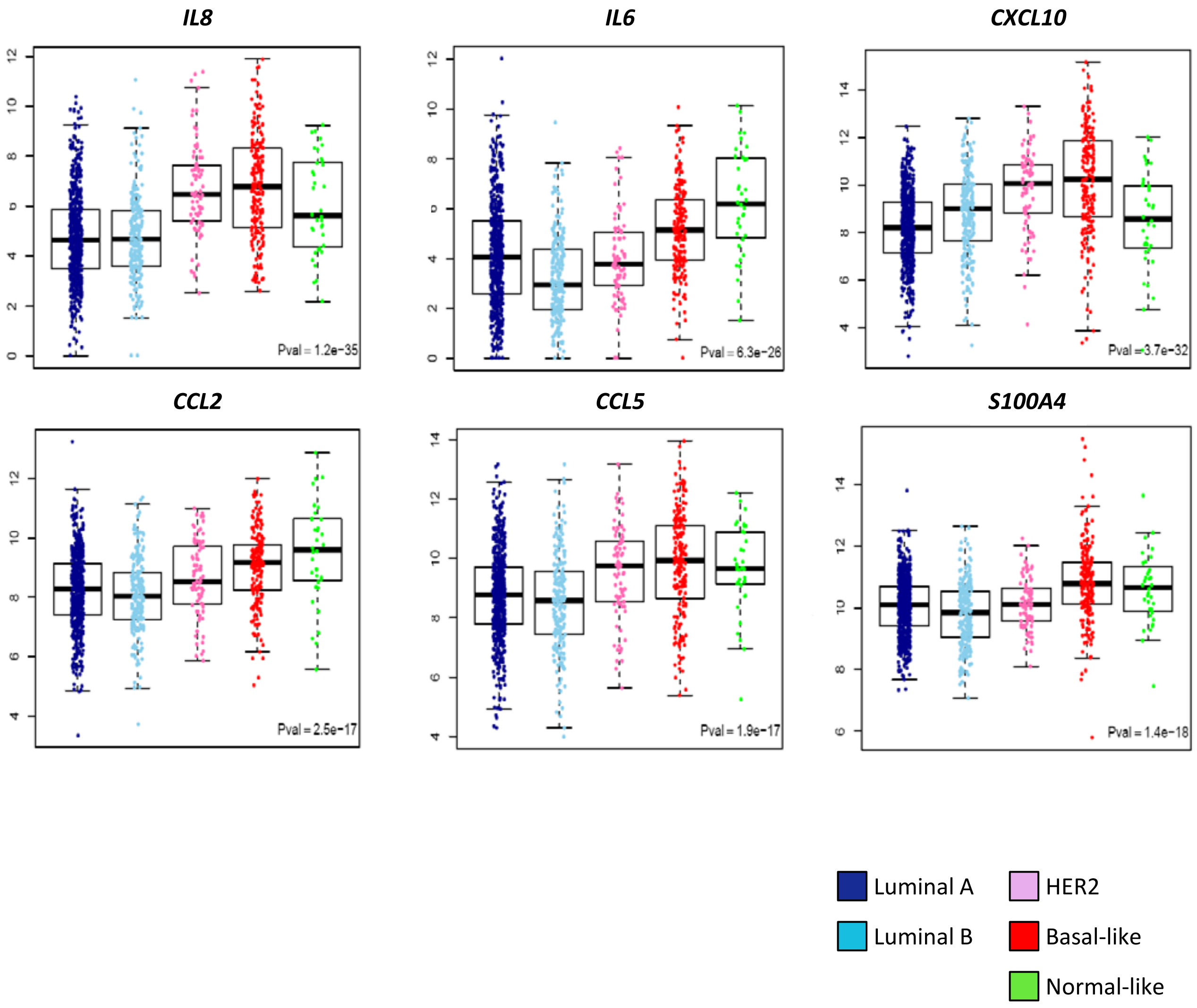

Supplementary Figure S2. Gene expression levels of the most abundant S100A4-inducible cytokines and S100A4 itself in the five molecular subtypes of BC from TCGAs collection of breast tumors. P-values indicated significant difference in expression between the subtypes. Kruskal-Wallis rank sum test was used to assess significant differences.

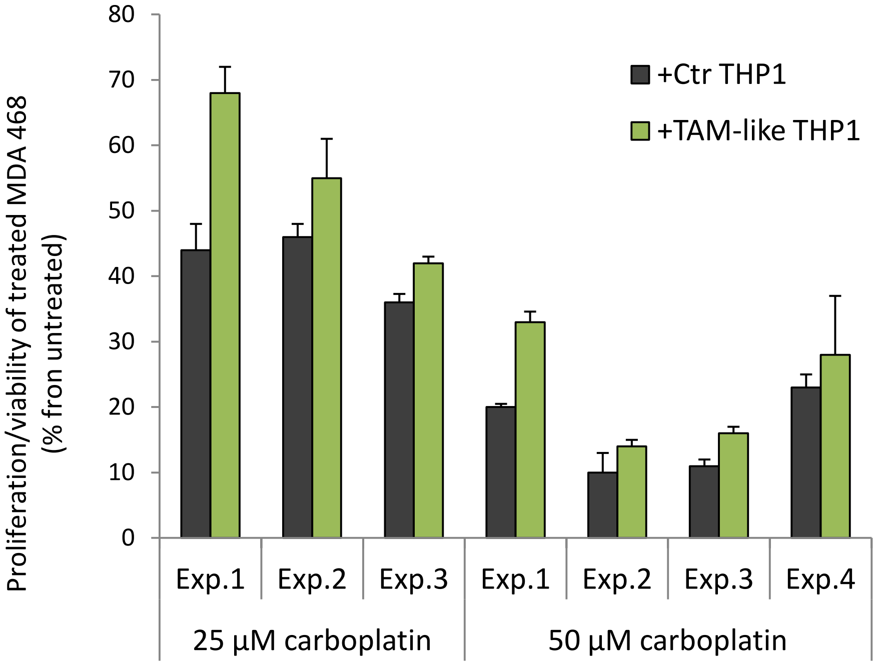

Supplementary Figure S3. TAM-like THP1 make MDA468 cells less sensitive to carboplatin. The co-cultures of GFP-Luc labeled MDA468 cells and Ctr THP1 or TAM-like THP1 were treated with 25 µM or 50 µM carboplatin for 3 days. The proliferation/viability of the treated cancer cells was scored by measuring Luc-mediated bioluminescence and is presented as % from respective untreated controls. The graph indicates average ± StDev from 3 technical paralells in each experiment (Exp.)

Supplementary Table S1. Primer sequences and probes used for qPCR.

Gene	Primer sequence	Universal probe
iNOS	forward: attcagctgtgccttcaacc<br>reverse: cattgccaaacgtactggtc	#66
CD206	forward: ggctacatatgccagacacg<br>reverse: ttaacaaagccatctgtttgaatc	#67
IL8	forward: agacagcagagcacacaagc<br>reverse: atggttccttccggtggt	# 72
IL6	forward: gatgagtacaaaagtcctgatcca<br>reverse: ctgcagccactggttctgt	#40
CCL2	forward: agtctctgccgcccttct<br>reverse: gtgactggggcattgattg	#83
CCL5	forward: cgctgtcatcctcattgcta<br>reverse: ggtgtggtgtccgaggaata	#16
CXCL10	forward: gaaagcagttagcaaggaaaggt<br>reverse: gacatatactccatgtagggaagtga	#34
CDH1	forward: gacacatttatggaacagaaaataaca<br>reverse: agtggaaatggcaccagtgt	#20
KRT19	forward: gccactactacacgaccatcc<br>reverse: caaacttggttcggaagtcat	#71
EPCAM	forward: ctccacgtgctggtgtgt<br>reverse: tgttttagttcaatgatgatccagta	#3
SNAI1	forward: gctgcaggactctaatccaga<br>reverse: atctccggaggtgggatg	#62
YARS (housekeeping)	forward: ggattaacaggcagcaaaatg<br>reverse: ccttccgatcaaggagatca	#35
RPL27 (housekeeping)	forward: cgcaaagctgtcatcgtg<br>reverse: gtcactttgcgggggtag	#83
